# Prader–Willi syndrome imprinting centre deletion mice have impaired baseline and 5-HT_2C_R-mediated response inhibition

**DOI:** 10.1093/hmg/ddz100

**Published:** 2019-05-14

**Authors:** Jennifer R Davies, Lawrence S Wilkinson, Anthony R Isles, Trevor Humby

**Affiliations:** 1 Behavioural Genetics Group, MRC Centre for Neuropsychiatric Genetics and Genomics, Neuroscience and Mental Health Research Institute, Schools of Medicine; 2 Psychology, Cardiff University, Cardiff, UK

## Abstract

Prader–Willi syndrome (PWS) is a neurodevelopmental disorder caused by deletion or inactivation of paternally expressed imprinted genes on human chromosome 15q11–q13. In addition to endocrine and developmental issues, PWS presents with behavioural problems including stereotyped behaviour, impulsiveness and cognitive deficits. The PWS genetic interval contains several brain-expressed small nucleolar (sno) RNA species that are subject to genomic imprinting, including *snord115* that negatively regulates post-transcriptional modification of the serotonin 2C receptor (5-HT_2C_R) pre-mRNA potentially leading to a reduction in 5-HT_2C_R function. Using the imprinting centre deletion mouse model for PWS (PWS*^ICdel^*) we have previously shown impairments in a number of behaviours, some of which are abnormally sensitive to 5-HT_2C_R-selective drugs. In the stop-signal reaction time task test of impulsivity, PWS*^ICdel^* mice showed increased impulsivity relative to wild-type (WT) littermates. Challenge with the selective 5-HT_2C_R agonist WAY163909 reduced impulsivity in PWS*^ICdel^* mice but had no effect on WT behaviour. This behavioural dissociation in was also reflected in differential patterns of immunoreactivity of the immediate early gene c-Fos, with a blunted response to the drug in the orbitofrontal cortex of PWS*^ICdel^* mice, but no difference in c-Fos activation in the nucleus accumbens. These findings suggest specific facets of response inhibition are impaired in PWS*^ICdel^* mice and that abnormal 5-HT_2C_R function may mediate this dissociation. These data have implications for our understanding of the aetiology of PWS-related behavioural traits and translational relevance for individuals with PWS who may seek to control appetite with the new obesity treatment 5-HT_2C_R agonist lorcaserin.

## Introduction

Prader–Willi Syndrome (PWS) is a neurodevelopmental disorder caused by deletion or inactivation of paternally expressed imprinted genes on human chromosome 15q11–q13 (1). The core phenotypic features of PWS are severe neonatal hypotonia and a failure to thrive in infancy, and the subsequent development of hyperphagia and reduced satiety responses result in obesity, unless managed carefully (2). Additionally, individuals with PWS also display a number of behavioural and cognitive problems. These are manifest as a mild to moderate intellectual disability with an average IQ of 60–80: impulsive and compulsive behaviours, including increased rates of tantrums, oppositionality and aggression (3) and a particular cognitive deficit in attention switching (4,5). Finally, a significant proportion of individuals with PWS will develop a severe affective psychotic illness (6). It is these behavioural and psychiatric problems that are most difficult to manage and affect quality of life for individuals and carers (7).

In the vast majority of cases of PWS, individuals have a genetic or epigenetic abnormality that leads to complete loss of all paternally expressed genes on 15q11–q13, including *MKRN3*, *MAGEL2*, *NDN*, *NPAP1, SNURF-SNRPN*, the C/D box small nucleolar (sno) RNAs *SNORD109A, SNORD115* and *SNORD116* (previously termed *HBII-438A, HBII-52* and *HBII-85,* respectively) and the non-coding RNA *IPW.* However, rare clinical cases have narrowed the critical PWS genetic interval to a region spanning the *SNORD116* repeat and the *IPW* gene (8–10). The importance of this interval is supported by animal studies showing paternal deletion of *Snord116* and *Ipw* leads to feeding, anxiety, sleep, growth and metabolic phenotypes reminiscent of PWS (11–14). However, although data is currently limited, deletion of *Snord116* and *Ipw* alone does not appear to contribute to other behavioural and cognitive problems (15). In contrast, using the PWS*^ICdel^* mouse model, in which an imprinting centre (IC) deletion results in complete loss of paternal gene expression at the PWS interval, we have demonstrated a wide range of behavioural abnormalities. In addition to feeding (16) and metabolic (17) phenotypes, PWS*^ICdel^* mice are hypoactive and show impaired sensory-motor gating (18). Furthermore, PWS*^ICdel^* mice show a number of cognitive abnormalities, including enhanced appetitive learning and reversal learning (19) and deficits in visuo-spatial attention (18).

By using the PWS*^ICdel^* mice we have also demonstrated that abnormal post-transcriptional processing of the serotonin 2C receptor (5-HT_2C_R) pre-RNA contributes to aspects of these behavioural phenotypes (20,21). The 5-HT_2C_R pre-RNA is subject to adenosine-to-inosine editing at five sites within an alternatively spliced exon, exon Vb. Both of these events result in less functional receptor moieties due to their effects on the amino acid sequence of the critical G-protein binding domain. Specifically, RNA-editing within exon Vb leads to a change in codon-specificity and subsequent amino acid sequence. Alternate splicing of exon Vb results in a truncated protein lacking a functional G-protein binding domain. Although this truncated splice variant cannot act as a receptor, it plays a critical role in overall 5-HT_2C_R function, by forming a heterodimer and sequestering the full-length splice variant in the endoplasmic reticulum and reducing cell surface expression (22). Processing of *Htr2c* pre-RNA is mediated, in part, by the actions of the snoRNA *Snord115* (23–25), present within the imprinted PWS locus (26). Expression of *Snord115* is lost in PWS*^ICdel^* mice (18), and there are concomitant increases in 5-HT_2C_R RNA editing (21) and splicing (20). Although loss of *SNORD115* alone does not give rise to PWS *per se* (27), PWS*^ICdel^* mice demonstrate abnormal 5-HT_2C_R-mediated feeding (20), suggesting that this snoRNA may contribute to the general PWS phenotype in those cases where all paternal gene expression is lost (28).

**Figure 1:**
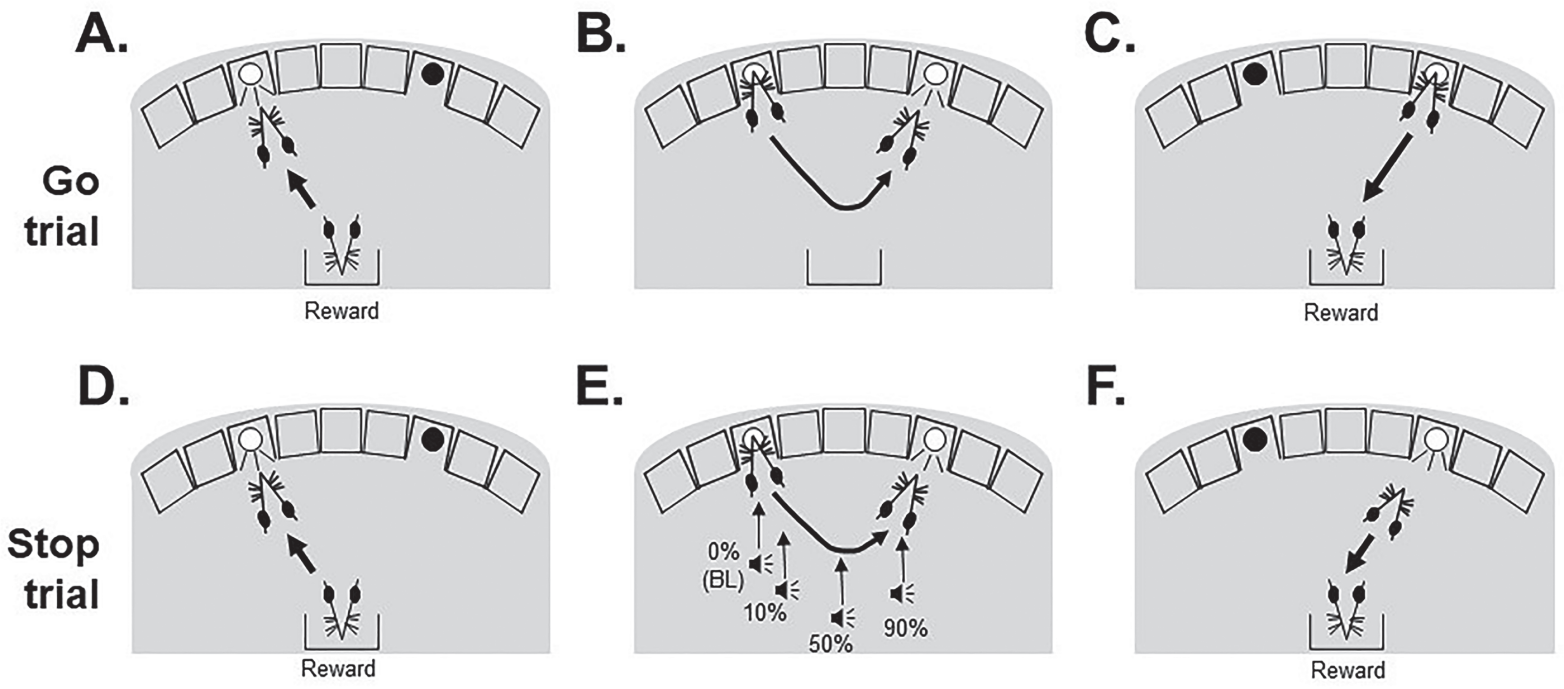
BL performance in the stop-signal reaction time task (SSRTT). Within each 100-trial session of the SSRTT, two types of trial were interpolated: go trials (80%, **A**–**C**) and stop trials (20%, **D**–**F**). Subjects initiated a trial with a nose-poke response to the ‘initiation’ stimulus in the left-hand hole. (B) If the trial was a go trial, then the go stimulus (light in right-hand hole) was illuminated, and a successful response at this location (C) would lead to delivery of 22 μL of the 10% condensed milk reward. The duration that the go stimulus was illuminated for was titrated for each individual subject, and failing to make a go response was recorded as an omission and resulted in 5 s time-out period signified by illumination of the house-light. Following collection of the reward, or a time out, the next trial was started by illumination of the initiation stimulus. (D) Stop trials were also initiated by a left nose poke to the initiation stimulus, which also subsequently resulted in presentation of a light in the right-hand hole (E). However, on these trials an auditory stop signal was also presented after trial initiation, instructing the mouse to inhibit responding to the second stimulus. (F) Successful stopping resulted in reward delivery on these trials. In separate test sessions, the stop signal was presented at different positions into a mouse’s individualized go reaction time, with early stop signals (0%) allowing for easier stopping, and later signals, close to the execution of the second stimulus response (90%), making stopping more difficult. Failing to stop (i.e. making the second nose poke) resulted in 5 s time-out period and a new trial starting.

The current study investigated the impact of abnormal 5-HT_2C_R functioning in PWS*^ICdel^* mice on a key aspect of cognition, namely response control. Specifically, we examined the performance of the PWS*^ICdel^* mice to inhibit a pre-potent response in a stop-signal reaction time (SSRT) task. The SSRT task (Fig. 1) is based on a ‘race’ between two response tendencies, ‘going’ and ‘stopping’ (29,30), where the subjects are trained to make a rapid go response, between two stimuli locations, but must withhold this response when a stop signal is presented. The closer the stop signal is to the initial movement the easier it is to stop the go response, and if the stop signal is presented towards the end of the go response, stopping is much more difficult. Performance of mice in the SSRTT is sensitive to pharmacological manipulation of 5-HT_2C_R (31). We found that PWS*^ICdel^* mice displayed baseline (BL) deficits in the ability to inhibit responding (i.e. increased impulsivity) compared to wild-type (WT) littermate controls. Furthermore, administration of a selective 5-HT_2C_R agonist had dissociable effects between PWS*^ICdel^* and WT mice, which was also reflected by specific alterations in the pattern of neuronal activity, as measured by c-Fos activation, in brain regions known to be important for correct response control (32). These data suggest a psychological basis to the impulsive/compulsive endophenotype shown by PWS patients. This abnormal response control may be mediated, in part, by aberrant 5-HT_2C_R functioning and therefore these findings have translational relevance for individuals with PWS who may seek to control appetite with the new obesity treatment 5-HT_2C_R agonist lorcaserin.

## Results

### No differences in shaping and training between WT and PWS^ICdel^ mice

PWS*^ICdel^* and WT mice were generated by crossing males positive for the IC deletion with CD1 females and consistent with previous studies (18,33), the PWS*^ICdel^* mice were considerably smaller than their WT littermates (31% and 30% lighter in weight, for males and females, respectively), although this size difference did not affect their propensity to consume reward during habituation to the reinforcement (Table 1). The majority of the mice were able to learn the SSRTT (Fig. 1), passing through the different stages of shaping and training (See Supplementary Material, Table S1); however, two WT and two PWS*^ICdel^* mice failed to reach criteria during training and were therefore removed from the study leaving a final *N* of 13 and 14 for the groups of WT and PWS*^ICdel^* mice, respectively (Table 1). There were no gender- or genotype-related differences in the number of sessions taken to reach BL SSRTT performance (Table 1), and there were also no differences in the mean programmed go stimulus duration (main effect of GENOTYPE, F_1,23_ = 3.64, *P* = 0.08, η^2^ = 0.14) or time that the mice were required to withhold a response in a stop trial to earn reward (main effect of GENOTYPE, F_1,23_ = 1.16, *P* = 0.29, η^2^ = 0.05), key parameters which govern performance in the SSRTT (Table 1).

### PWS^ICdel^ mice show more impulsivity than WT littermates in the SSRTT

**Figure 2:**
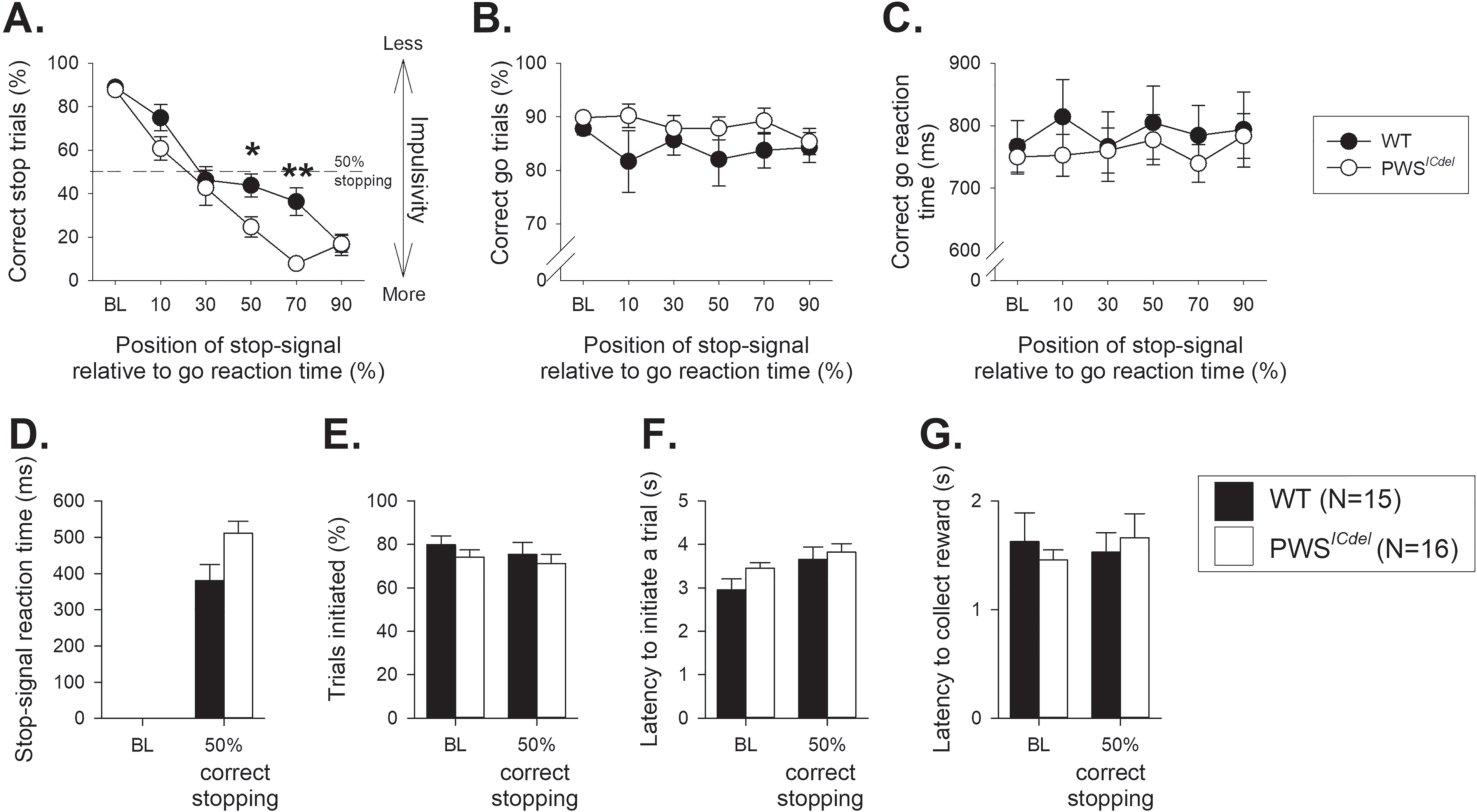
Stop-signal reaction time task performance in PWS*^ICdel^* mice. (**A**) Altering the position of the auditory stop-signal led to the anticipated increase in impulsive responding as stopping was made more difficult with presentations progressively closer to the execution of the go response. However, PWS*^ICdel^* mice showed increased impulsivity relative to WT littermate controls, particularly when the stop signal was presented near the middle of the individual go response. (**B**) Altering the position of the stop signal did not affect the proportion of correct go responses made or the speed of the go response (**C**). However, there was a tendency for PWS*^ICdel^* to make more correct responses than their WT littermates. Further analysis of SSRTT performance was conducted to examine the point where competition between the go and stop responding was at its greatest; thus, data from each subject were ranked and sessions where correct stopping was at 50 ± 10% averaged. When stopping with 50% accuracy, the SSRT, the latency to withhold responding, was calculated (**D**). PWS*^ICdel^* mice had significantly longer SSRTs than their WT littermates, but there were no genotype-related differences for the proportion of trials initiated (**E**) and increases in the latency to initiate a trial (**F**) and the time taken to enter the food magazine following a successful trial (**G**). Baseline data (BL: i.e. mean of the sessions immediately preceding each session where the stop-signal position was altered) when the stop-signal presentation was concurrent with the start of the go response (0%). Data are mean ± SEM, ^*^ and ^**^ denotes *P* < 0.05 and ^**^P < 0.01 for significant difference between WT and PWS*^ICdel^* mice, respectively.

All of the mice showed the anticipated increase in impulsive responding as the stop-signal was moved closer to the end of the go response, represented by the reduced ability to stop and withhold responding (Fig. 2A, main effect of STOP-SIGNAL POSITION, DELAY, F_5,125_ = 67.65, *P* = 0.001, η^2^ = 0.73); however, PWS*^ICdel^* mice showed increased impulsivity relative to WT littermate controls indicated by decreased proportion of correctly stopped trials (main effect of GENOTYPE, F_1,25_ = 4.74, *P* = 0.04, η^2^ = 0.16). A significant STOP-SIGNAL POSITION^*^GENOTYPE interaction (F_5,125_ = 2.38, *P* = 0.04, η^2^ = 0.09) indicated that PWS*^ICdel^* mice differed from WT, only when the stop-signal was presented between 40% and 70%, or halfway through the individualized go response (*post hoc* analysis for each stop-signal position: 40% *P* = 0.002, 50% *P* = 0.11, 60% *P* = 0.033, 70% *P* = 0.001), the point where the stop and go processes are competing at their greatest. There were no differences between PWS*^ICdel^* and WT mice in the number of correct go responses (Fig. 2B, main effect of GENOTYPE, F_1,25_ = 1.36, *P* = 0.25, η^2^ = 0.05), or in the speed of making the go response (Fig. 2C, main effect of GENOTYPE, F_1,25_ = 0.23, *P* = 0.64, η^2^ = 0.01). Altering the stop-signal position on ‘stopping’ were quite specific, as these manipulations did not affect amount of (main effect of STOP-SIGNAL POSITION, F_5,125_ = 0.40, *P* = 0.85, η^2^ = 0.02), or the latency in these interpolated go trials (main effect of STOP-SIGNAL POSITION, F_5,125_ = 1.19, *P* = 0.32, η^2^ = 0.05).

**Table 1:** Task shaping and comparison of gender during training and at with 50% stopping ability

		Number of		Shaping and training		Performance at 50% correct stopping					
				subjects						Going			Stopping		
		Start	At BL	Sessions to BL	Sessions to stable BL performance	Duration of go stimulus (ms)	Correct responses (%)	Reaction time (ms)	Time to withhold responding (ms)	Correct responses (%)	SSRT (ms)
*WT*	Male	4	4	44.25 ± 1.65	13.75 ± 5.17	1475.00 ± 137.69	78.31 ± 3.23	877.78 ± 49.27	612.50 ± 42.70	52.76 ± 3.24	450.15 ± 63.17
	Female	11	9	37.22 ± 2.38	13.78 ± 3.22	1100.00 ± 27.39	84.55 ± 5.59	718.58 ± 52.19	533.33 ± 37.91	46.96 ± 1.22	349.50 ± 56.60
	All WT	15	13	39.38 ± 2.62	13.77 ± 2.62	1215.38 ± 65.87	82.63 ± 3.99	798.33 ± 51.35	557.69 ± 31.44	48.74 ± 1.45	380.47 ± 44.33
PWS*^ICdel^*	Male	10	9	43.56 ± 0.80	8.11 ± 1.97	1134.72 ± 91.52	85.58 ± 3.13	744.50 ± 54.24	583.33 ± 47.14	50.22 ± 1.50	499.95 ± 39.86
	Female	6	5	41.60 ± 0.80	11.80 ± 3.26	1120.00 ± 60.42	90.75 ± 2.73	751.65 ± 36.64	670.00 ± 46.37	46.31 ± 4.56	531.74 ± 64.98
	All PWS*^ICdel^*	16	14	42.86 ± 0.65	9.43 ± 1.72	1129.46 ± 60.98	87.43 ± 2.27	747.05 ± 36.23	614.29 ± 35.33	48.82 ± 1.86	511.30 ± 33.32
											
WT: GENDER comparison				*P* = 0.098	*P* = 0.421	*P* = 0.069	*P* = 0.354	*P* = 0.064	*P* = 0.212	*P* = 0.171	*P* = 0.270
PWS^ICdel^ GENDER comparison				*P* = 0.156	*P* = 0.365	*P* = 0.895	*P* = 0.238	*P* = 0.915	*P* = 0.217	*P* = 0.453	*P* = 0.689

Notes: Sessions to BL reflect the number of sessions taken to progress through the first stages of training (See Supplementary Material, Table S1), and sessions for stable BL performance the number of sessions for consistent intra-session performance in the full SSRTT with stop-signal presentation coincident with the start of the go response (i.e. a 0% stop-signal delay). Other measures are from when individualized performance was assessed over the range of stop-signal positions used (10%, 20%, 30%, 40%, 50%, 60%, 70%, 80% and 90% of the go response) and 50% stopping was calculated. Separate unpaired two-tailed *t*-tests were used for the gender comparisons for each genotype. Data shows mean ± SE. SSRT: stop-signal reaction time (see main text for derivation).

Examination of these effects further, by looking at the point where the mice were making 50% correct stop responses (see Materials and Methods), showed that for PWS*^ICdel^* mice, the stop signal was presented 30.4 ± 5.5% into the go response, compared with 46.7 ± 6.6% for their WT littermates. At this point, the SSRT, the virtual time take to decide to stop responding can be calculated, which was significantly slower for PWS*^ICdel^* mice in comparison to WT (Fig. 2D, t_25_ = 2.38, *P* = 0.025), consistent with a pattern of more impulsive responding. There were no differences between PWS*^ICdel^* and WT mice for ancillary task measures, such as the proportion of trials started (Fig. 2E, main effect of GENOTYPE, F_1,25_ = 0.14, *P* = 0.71, η^2^ = 0.01), time taken to start a trial (Fig. 2E, main effect of GENOTYPE, F_1,25_ = 2.56, *P* = 0.12, η^2^ = 0.09) or for the latency to collect the reward (Fig. 2E, main effect of GENOTYPE, F_1,25_ = 1.85, *P* = 0.19, η^2^ = 0.07), further demonstrating the specificity of the effects on stopping.

The effects of gender on SSRTT performance were also examined, especially in terms of interactions with the genotype of the mice. Although, there were some significant effects of GENDER and interactions between GENOTYPE and GENDER, these were derived from differences between male and female WT mice and there were no differences between male and female PWS*^ICdel^* mice (See Supplementary Material, Table S1).

### Effects of 5-HT_2c_R agonism on SSRTT performance

With increased alternate splicing (20) and RNA-editing (21) of 5-HT_2C_R RNA in the brains of PWS*^ICdel^* mice, and the demonstration that manipulating 5HT_2C_R can affect stopping in the SSRTT (31) we investigated whether PWS*^ICdel^* mice would show differential affects to WT in response to WAY163909, a specific 5-HT_2C_R agonist. The effects of WAY163909 were investigated in sessions were the stop signal was placed at a point in the individualized go responses where each mouse had shown 50% correct stopping, i.e. the point where the go and stop responses were equivalent. For the parameters analysed there were no main effects of GENDER, or interactions between GENOTYPE and GENDER in the initial ANOVA with a planned contrast to vehicle treatment (See Supplementary Material, Table S2), therefore the data were re-analysed without GENDER as a factor.

**Figure 3:**
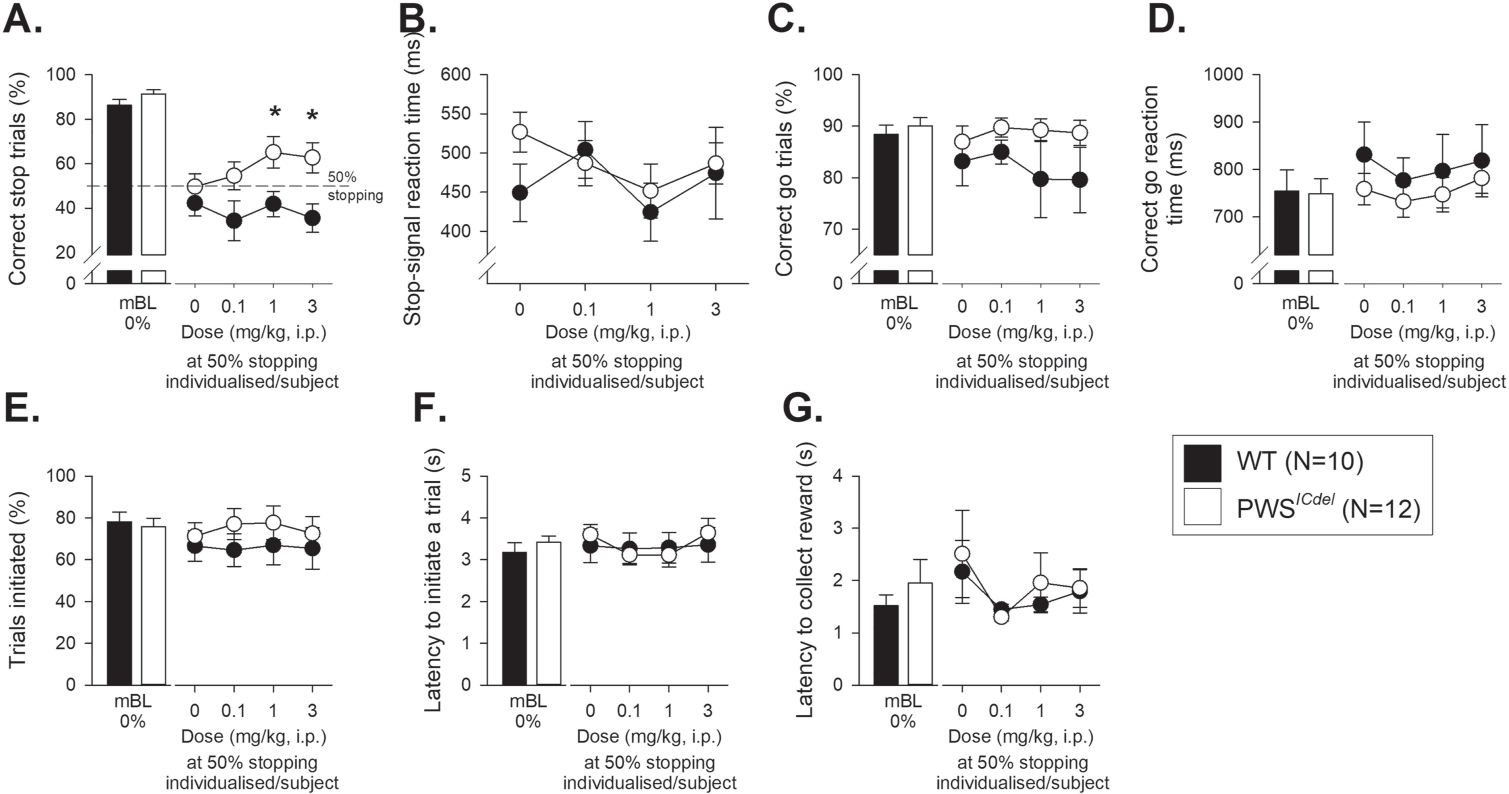
Effects of the 5HT_2c_ agonist WAY163909 administration on stop-signal reaction time task performance in adult PWS^ICdel^ mice, injected at point where the individual mice demonstrated 50% stopping. At all doses used, WAY163090 improved stopping performance in PWS*^ICdel^* mice, but did not affect stopping behaviour of WT littermates (**A**); however, the SSRT (**B**) was unaffected. The specificity of WAY163909 to affect stopping behaviour was shown by a lack of effects on the proportion of correct go trials (**C**), speed of the go response (**D**), the proportion of trials performed (**E**), the latency to initiate a trial (**F**) and the latency to collect reward (**G**). Baseline data (mBL: i.e. mean of the five sessions immediately preceding each drug treatment session) when the stop-signal presentation was concurrent with the start of the go response (0%) are shown for illustrative purposes and were not included in the statistical analysis. Data shows mean ± SE, ^*^ denotes *P* < 0.05 for compassion between WT and PWS*^ICdel^* mice.

WAY163909 significantly increased the ability of PWS*^ICdel^* mice to withhold responding under conditions where stopping was at 50% (Fig. 3A, main effect of GENOTYPE, F_1,20_ = 6.94, *P* = 0.016, η^2^ = 0.26). A significant interaction for the linear contrast between DOSE and GENOTYPE (F_1,20_ = 6.09, *P* = 0.023, η^2^ = 0.23) demonstrated that this effect occurred only at the higher does used, confirmed by *post hoc* comparisons between WT and PWS*^ICdel^* mice (t_20_ = 0.12, t_20_ = 0.02 and t_20_ = 0.014 at the 0.1, 1 and 3 mg/kg doses, respectively). WAY 163909 at all doses did not affect stopping behaviour in the WT mice, where performance remained consistent with vehicle treatment. WAY163909 did not significantly alter any other of the task parameters and there were no genotype-related effects of the drug at any dose on the SSRT (Fig. 3B, main effect of GENOTYPE, F_1,20_ = 0.41, *P* = 0.53, η^2^ = 0.02), proportion of go responding (Fig. 3C, main effect of GENOTYPE, F_1,20_ = 1.65, *P* = 0.21, η^2^ = 0.08), speed of the go response (Fig. 3D, main effect of GENOTYPE, F_1,20_ = 0.66, *P* = 0.43, η^2^ = 0.03), proportion of trials started (Fig. 3E, main effect of GENOTYPE, F_1,20_ = 1.09, *P* = 0.31, η^2^ = 0.05), trial initiation latency (Fig. 3F, main effect of GENOTYPE, F_1,20_ = 0.02, *P* = 0.88, η^2^ = 0.01) or the latency to collect the reward (Fig. 3G, main effect of GENOTYPE, F_1,20_ = 0.13, *P* = 0.83, η^2^ = 0.01).

### Abnormal c-Fos immunoreactivity in PWS^ICdel^ mouse brains

**Figure 4:**
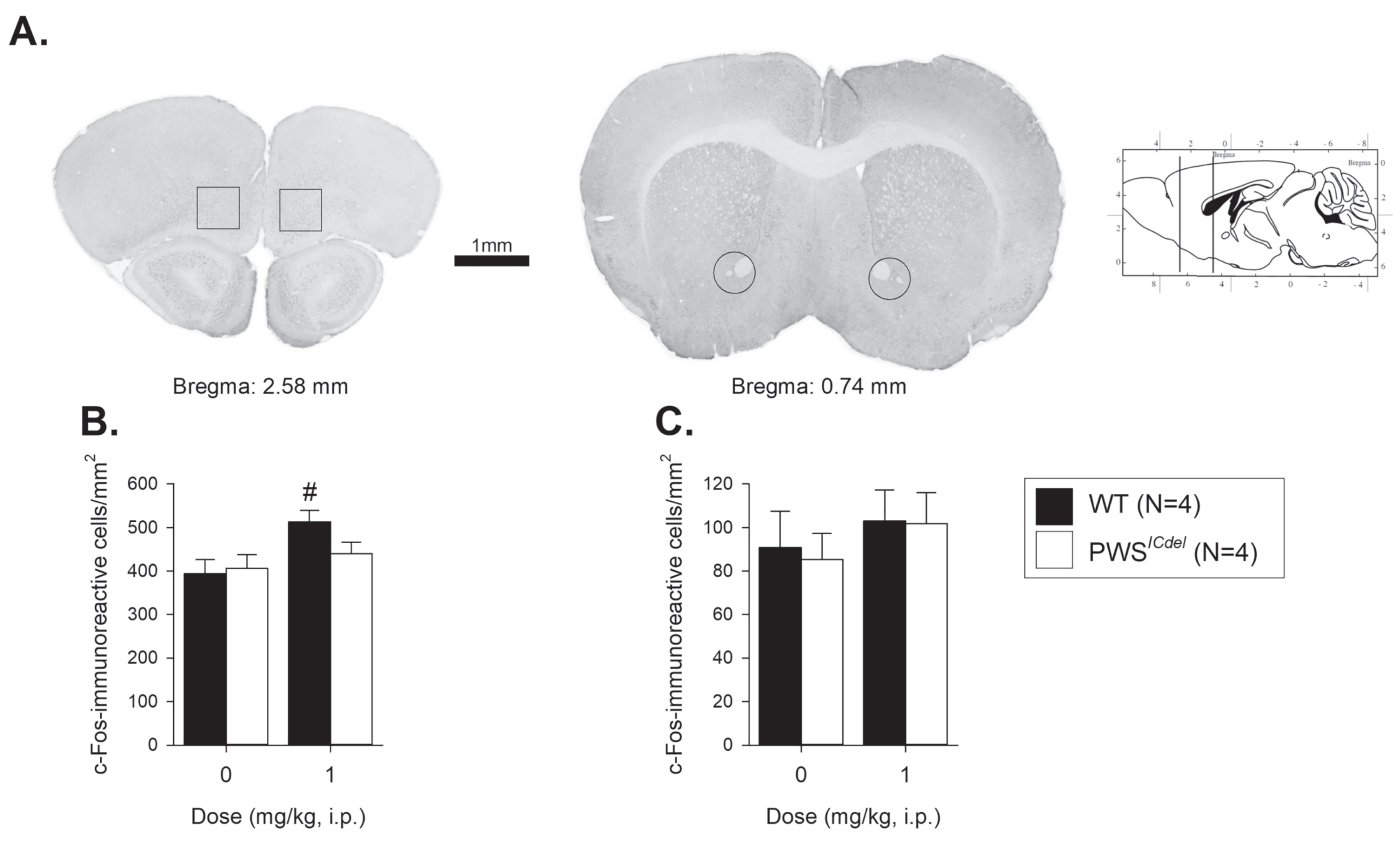
Effects of the 5HT_2c_ agonist WAY163909 administration on c-Fos immunoreactive cells in adult PWS*^ICdel^* mice. c-fos reactivity was assessed in two brain regions, the OFC and the nucleus accumbens (**A**). 1 mg/kg WAY163909 significantly increased the number of c-Fos positive cells in the OFC in WT, but not PWS*^ICdel^* mice (**B**). WAY163909 did not affect the number of c-Fos positive cells in the nucleus accumbens, for either WT pr PWS*^ICdel^* mice (**C**). Data shows mean ± SE. # denotes *P* < 0.05 for comparison of vehicle to WAY163909 for each group.

Injection of WAY163909 (1 mg/kg, i.p.) resulted in differential effects on c-Fos reactivity between PWS*^ICdel^* and WT littermate mice that were specific to the brain regions analysed (Fig. 4A and B). In the orbitofrontal cortex (OFC), the 5-HT_2c_ agonist significantly increased the number of c-Fos positive cells (Fig. 4B, main effect of DOSE, F_1,12_ = 6.85, *P* = 0.02, η^2^ = 0.36), in both WT and PWS*^ICdel^* mice (main effect of GENOTYPE, F_1,12_ = 1.07, *P* = 0.32, η^2^ = 0.08). However, this increase was greater in WT mice (~23% increase relative to ~ 8% increase in PWS*^ICdel^* mice, t_6_ = 1.96, *P* = 0.09), with only a significant increase in c-fos reactivity in WT OFC samples (t_6_ = 2.84, *P* = 0.03, cf. PWS^ICdel^: t_6_ = 0.84, *P* = 0.44, for comparison between vehicle and WAY163909 treatment). In the nucleus accumbens, there was no effect of WAY163909 on c-Fos reactivity (Fig. 4C, main effect of DOSE, F_1,12_ = 0.99, *P* = 0.34, η^2^ = 0.08) in either PWS*^ICdel^* mice or their WT littermates (main effect of GENOTYPE, F_1,12_ = 0.06, *P* = 0.82, η^2^ = 0.01).

## Discussion

We show that PWS*^ICdel^* mice have impaired response inhibition, as indicated by a reduced ability to stop in the SSRTT, and an increased correct SSRT. Given previous work indicating increased alternate splicing and RNA-editing of *Htr2c* in the PWS*^ICdel^* mice and the role of 5-HT_2C_Rs in response inhibition, we further explored stopping performance by systemic administration of a 5-HT_2C_R agonist (WAY163909). PWS*^ICdel^* mice demonstrated an abnormal response relative with WT littermate controls. This behavioural response to 5-HT_2C_R agonism was also reflected in blunted c-Fos activation in the orbital frontal cortex of the PWS*^ICdel^* mice following 5-HT_2C_R agonist. These data suggest impaired response control, mediated in part by aberrant 5-HT_2C_R functioning, could be a psychological basis to the impulsive/compulsive endophenotype shown by PWS patients.

Consistent with our previous studies (31,34), all mice were able to complete the SSRTT and demonstrated the expected patterns of behaviour, with stopping performance systematically decreasing as the stop-signal moved closer to the end of the go reaction time. There were no effects of stop-signal position on the go correct rate, or correct go reaction time. However, PWS*^ICdel^* mice showed reduced response inhibition (i.e. increased impulsivity) in the SSRTT as indexed by impaired stopping accuracy and longer SSRT (the time taken after a stop-signal is presented for inhibition to be completed), the standard measure of response inhibition. SSRT cannot be measured directly as there is no observable endpoint to the response inhibition but is estimated using a well-defined and tested mathematical model, the race model (30).

In addition to a BL measure of response control, we also wanted to probe the function of the serotonin system in PWS*^ICdel^* mouse model while performing the SSRTT. In particular, we focused on the serotonin 2C receptor (5-HT_2C_R). This is because post-transcriptional modification of *Htr2c* RNA is altered in the brain of PWS*^ICdel^* mice, with increased alternate splicing (20) and RNA-editing (21), most probably as a consequence of loss of brain-specific snoRNA *snord115* in this model. Furthermore, there is a known role for 5-HT_2C_R in stopping behaviour, as response control is improved by the 5-HT_2C_R-specific antagonist SB242084 in the SSRTT (31). Here we employed the use of the 5-HT_2C_R-specific agonist WAY163909 and saw a divergence in effects on stopping behaviour between WT and PWS*^ICdel^* mice. Specifically, following WAY163909 administration WT mice showed some reduction in performance but were generally unaffected by the drug, whereas PWS*^ICdel^* mice displayed a marked improvement in stopping behaviour. Although not as clear-cut, this pattern of effects is very similar to those seen on feeding behaviour, where 3 mg/Kg WAY163909 inhibited feeding in WT mice, but enhanced feeding in PWS*^ICdel^* mice (20). Moreover, this apparent opposite effect of WAY163909 administration, decreasing impulsivity in the SSRTT and increasing feeding, may be a result of effects of 5-HT_2C_R agonism in different parts of the brain with distinct dissociable functions on behaviour. However, a recent study (35) has demonstrated that agonism of the 5-HT_2C_R with WAY163909, affected response control, akin to the improved stopping we have found here, in combination with consumption of a high calorific diet suggesting a relationship between dietary habits and decision-making strategies with 5-HT_2C_R at the nexus (36,37).

The pattern of findings of impaired response control on the SSRTT contrasts with previous findings in the five-choice serial reaction time task (5-CSRTT), where PWS*^ICdel^* mice exhibited no differences in premature responding (21). The 5-CSRTT is primarily used to measure visuospatial attention, and indeed PWS*^ICdel^* mice were found to be impaired in attentional function (18). However, premature responding in the 5-CSRTT is also a measure of response inhibition (38). Why PWS*^ICdel^* mice show BL deficits in one task and not the other could be due two possible reasons. First, it could be that PWS*^ICdel^* mice are generally impaired in response control, but that the tasks are not equivalent in terms of difficulty and so BL deficits only emerge on the more demanding SSRTT where the inhibitory load is highest (39). Alternatively, the PWS*^ICdel^* mice may be more impaired in a specific aspect of response control. It is now established that response control can be dissociated into a number of psychologies that are also neurally distinct (40,41). This is certainly the case for the SSRTT and the 5-CSRTT that measure the ability to cancel an ongoing action (‘stopping’) and the ability to inhibit the initiation of an action (‘waiting’), respectively. Our data would therefore suggest that PWS*^ICdel^* mice are more impaired at stopping (an ongoing action), something that could, in part, be due to the differential influence of less-functional 5-HT_2C_Rs in this model.

To further explore the idea that 5-HT_2C_R function varies in the PWS*^ICdel^* mice, we also examined c-Fos activity in the brain following administration of 1 mg/Kg WAY163909. This dose was selected as it resulted in the biggest divergence in stopping behaviour between PWS*^ICdel^* and WT mice. Thus, following drug treatment, PWS*^ICdel^* mice showed reduced c-Fos immunoreactivity in the OFC, an area that is important for ‘stopping’ behaviour (42) but not in the nucleus accumbens, an area that is less critical for inhibitory behaviour in the SSRTT (43,44). This blunted engagement of OFC c-Fos neurons may be a reflection of diminished activation of 5-HT_2C_R in PWS*^ICdel^* mice, as a result of a decrease of the most active 5-HT_2C_R isoforms in this area. Regional variation in the degree of post-transcriptional modification, giving rise to differential sensitivity and activation, has been reported in the rat brain (45), and it may well be that this is reflected in variability of 5-HT_2C_R isoform expression, and therefore drug sensitivity, in PWS*^ICdel^* mice. Regional variation in 5-HT_2C_R functionality, and recruitment of different brain regions implicated in response control, may also account for the dissociation in altered impulsive responding in PWS*^ICdel^* mice between the current data using the SSRTT, and previous findings in the 5-CSRTT (18).

Taken together with previous findings, this study suggests that response control is compromised in PWS*^ICdel^* mice. This cognitive deficit may underpin some of the behavioural characteristics seen in PWS, such as temper tantrums, stubbornness and compulsive behaviour (3). The work also adds to the burgeoning literature suggesting that 5-HT_2C_R function is impaired in the PWS*^ICdel^* mice (20,21). The most logical explanation for this is alteration in alternate splicing and/or editing of 5-HT_2C_R pre-mRNA due to loss of expression of the imprinted regulatory snoRNA *snord115* (20,21,23). However, it should also be noted that serotonin neurochemistry is also altered in mice that lack the PWS imprinting locus genes *Necdin* (46) or *Magel2* (47) alone. Nevertheless, the specificity of some the effects demonstrated here and previously (21) indicates these are not just generalized abnormalities due to serotonin imbalance but are due, in part, to global impairments in 5-HT_2C_R function in the PWS*^ICdel^* mice. With the introduction of 5-HT_2C_R agonists, such as lorcaserin, to control appetite and act as anti-obesity treatments (48), our results have translational implications for treatment of individuals with PWS. As demonstrated in our current and previous studies (e.g. 21), using 5-HT_2C_R agonists may produce differential effects in PWS patients to non-affected people, therefore management of PWS-related dietary issues using these drugs may not be less affective and alternative therapeutic pathways may need to be pursued.

## Materials and Methods

### Subjects

Sixteen PWS*^ICdel^* and fifteen WT littermates adult mice, aged ~ 4 months old at the start of the experiment were used. Mice were obtained from breeding males positive for the IC deletion with CD1 females, which generated paternally derived mutants and WT littermates; the nature of the epigenetic regulation of imprinted genes means that a paternally inherited IC deletion will result in a lack of gene expression from the PWS interval. As previously described (18,21,33) breeding to a C57BL/6 J*CD1 background reduces the risk of postnatal lethality in PWS*^ICdel^* mice; however, to increase yield excess WT pups in each litter were culled (identified on the basis of their increased size 48 h after birth) leaving only one or two per litter. However, even with these measures in place, both male and female mice were used to generate a viable amount of PWS*^ICdel^* mice for the current study, housed in separate sex litters with two to three animals each. All subjects were housed in a vivarium with environmental control (temperature: 21 ± 2°C, humidity 50 ± 10%) and a 12:12 light/dark cycle (lights on at 07:00 hr). Following 2 weeks of habituation and handling, a home cage water and food restriction schedule was introduced systematically for over a further 2 weeks. For the duration of the experiment the mice received access to water and food for 2 and 8 h access/day, respectively. This regime maintained the subjects at ~ 90% of free-feeding body weight and motivated the animals to work in the task. All procedures were conducted in accordance with the requirements of the UK Animals (Scientific Procedures) Act 1986 and with local institute approval from the School of Psychology, Cardiff University.

### Apparatus

The SSRTT was performed in mouse nine hole chambers (31,49) (Campden Cognition, UK) with the task under the control of custom written software. Each test chamber (14 × 13 cm) was enclosed in a sound attenuating box equipped with a fan to provide ventilation and also a consistent level of background noise. The test chamber was equipped with nine circular response apertures (10 mm diameter) arranged in an arc on the back wall. Each aperture was configured with a vertically orientated infrared beam and a 40 mA stimulus light at the distal end. For the SSRTT the stimulus array was configured such that only two of the stimuli apertures were open (the others were blocked by black plastic). The open apertures, holes number 3 and 7 (from the left), were equally placed relative to the centre line of the chamber and were designated as the initiation and go responses, respectively. The near wall, including the access door, held the food magazine (2 cm wide) that was enclosed by a clear Perspex door and could also be illuminated by a 60 mA lamp. Opening of the food magazine door was recorded by the triggering of a micro-switch, as panel pushes. Reward was delivered into a small well in the floor of the food magazine via a 21-gauge hypodermic needle and 0.8-mm silicone tubing from a peristaltic pump located outside of the test chamber but within the sound attenuating box. A 60-mA house light and speaker were fitted to both side walls of the test chamber and a pair of infrared beams which spanned the chambers, perpendicular to the stimulus array and 5 mm above the grid floor, were used to record motor activity. An infrared camera (Watac, USA) mounted inside the sound attenuating box permitted observation and recording of behaving mice. The white noise stop signal, 105 db, was provided by a custom built sound generator.

### SSRTT: initial shaping and training to BL

After the 2 weeks on the home cage water and food restriction schedule, the mice were habituated to the liquid reward to be used in the experiments, 10% condensed milk (Nestle Ltd, UK) solution, using methods employed previously (31,49). All sessions in the nine-hole boxes were performed with the house light off. The subjects were first habituated to the chambers for 3 days before training to the BL SSRTT (Fig. 1), which involved shaping the mice to respond sequentially at two stimulus locations, using nose pokes, to give rise to a ‘go’ response, and then learning to withhold responding to the second stimulus location when an auditory stop signal was presented, to give rise to a ‘stop’ response (see Supplementary Material, Fig S1). During training, the duration of the go stimulus, and the time that a response had to be withheld in a stop trial, were determined individually for each subject. In an SSRTT session (≤20 min session duration), there was a maximum of 100 trials, where 80% of trials were ‘go’ trials and 20% were interpolated ‘stop’ trials (Fig. 1).

### SSRTT; behavioural manipulations

At BL, in ‘stop’ trials, the stop signal was always presented coincident with the beginning of the go response (i.e. 0% of the individual correct go reaction time for each subject), making stopping relatively easy, but in separate probe sessions the position of the stop-signal was varied relative to the individual correct go reaction times for each mouse making stopping more or less difficult i.e. at 10%, 20%, 30%, 40%, 50%, 60%, 70%, 80% and 90% into the individualized go reaction time for each mouse. At 90%, the stop signal was played close to the execution of the response, and hence stopping was most difficult (31,50). The order of presentation of these sessions was randomized between subjects using a Latin square design.

### Pharmacological administration for assessing SSRTT performance

The effects of the 5-HT_2C_R agonist WAY163909 ((7*bR*,10*aR*)-1,2,3,4,8,9,10,10*a*-Octahydro-7*bH*-cyclopenta-[*b*][1,4]diazepino[6,7,1hi]indole) (Sigma, UK) on SSRTT performance in WT and PWS*^ICdel^* mice were investigated using doses shown previously to have a biological effect in rodents (0, 0.1, 1 and 3 m/kg, (20,51)). Following at least 4 days of stable drug-free performance at BL SSRTT criteria (i.e. stop-signal position of 0%), intraperitoneal injections of vehicle or WAY163909 (0.1, 1 and 3 m/kg) made up freshly on the day of use as freebase in physiological saline were administered 30 min before testing following a Latin square design. Drug treatment was given in combination with SSRTT sessions where the stop-signal was presented at a position that had given rise to 50% correct stopping for each subject, thus individual stop-signal positions were employed for each subject. For these studies 10 WT and 12 PWS*^ICdel^* mice from the original cohort were used.

### Pharmacological administration for assessing c-Fos reactivity

On completion of the behavioural study, some of the WT and PWS*^ICdel^* mice were randomly assigned to receive either vehicle or 1 mg/kg WAY163909 i.p. treatment (four subjects/treatment/genotype, see Table 1) 90 min prior to sacrifice. During this interval, the animals remained uninterrupted in their home cage to permit optimum c-Fos expression. They were then deeply anaesthetized with Euthasol (100 mg/kg, i.p.) and perfused transcardially with 50 mL of ice-cold 0.1 M phosphate-buffered saline (PBS), pH 7.4, followed by 100 mL of 4% paraformaldehyde in 0.1 M PBS, pH 7.4. The brains were then removed, postfixed overnight at 4°C in 4% paraformaldehyde, and then transferred to 30% sucrose for 24 h while continuously being stored at 4°C. Coronal sections (40 μm) were collected using a freezing microtome then stored at −20°C in a cryoprotectant solution until required. Sections were first incubated in rabbit anti-Fos polyclonal antibody (ABE457; 1:3000; Merck) for 24 h at 4°C. Sections were then rinsed in TBST (3 × 10 min) containing 3% NGS, then incubated in Tris buffered saline with Tween-20 (TBST) containing biotinylated goat anti-rabbit IgG (1:100; Vector Laboratories) and 3% NGS for 1 h and then rinsed in 0.1 M TBST (3 × 10 min). After which sections were incubated for 1 h in ABC complex (Vectastain Elite ABC Kit; Vector Laboratories), then washed in 0.1 M TBST (3 × 10 min), then washed in 0.05 M Tris buffer (2 × 10 min). Sections were then incubated in DAB solution (DAB Peroxidase (HRP) Substrate Kit (with Nickel), 3,3′-diaminobenzidine; Vector Labs) for ~30 s. Sections were then mounted onto subbed slides, dried and dehydrated before cover slipping. Regions of interest, the OFC and nucleus accumbens, were selected according to a mouse stereotaxic atlas of the brain (52) (Fig. 4A and B). Three sections per brain region per animal were counted and averaged to give number of Fos positive cells within an area of 0.6 mm2 at 10× magnification using the Image J analysis program (version 1.43q, NIH, USA).

### Statistical analysis

The main measures from the SSRTT were the proportions of correct stopping and going, the correct go reaction time, the trial initiation latency, proportion of trials started and the reward collection latency. Ancillary measures of general task performance, including the numbers of food magazine entries, initiation and go nose-pokes and locomotion measured as beam break were also assessed. SSRTs in the task were estimated employing the standard procedure described in Logan *et al*. (30), using data from where the proportion of correct stop responses is ~ 50%. For each subject, data from the sessions in which the stop-signal positions were varied relative to the individualized go reaction time, were ranked by the proportion of correct stop responses, and data from sessions in which this value was between 40% and 60% (i.e. 50% ± 10%) were averaged. The latency of stopping as defined by the SSRT was derived from the distribution of correct go reaction times and the proportion of correctly stopped trials as previously described (29,53). Hence, for each of the sessions determined above, the correct go reaction times were rank ordered from smallest to largest and the nth value found, where *n* is the rank order position based on the proportion of failing to stop correctly in stop trials, corrected for the occurrence of omitted go trials (53–55). To determine the SSRT, the time the stop signal was presented (i.e. ‘mean correct go reaction time’ × ‘% mean stop-signal position’) was subtracted from the *n*th correct go reaction time value. Scores calculated as percentages were arcsine, and latencies square root transformed prior to analysis.

Data were analysed using SPSS (V.20, SPSS Inc, USA), and were first assessed for normality, and then subjected to two-tailed *t*-test or ANOVA, if appropriate or equivalent non-parametric analyses, and where sphericity assumptions were violated, Greenhouse–Geisser corrections were used. As both male and female mice were used in these experiments, all data was subjected to an initial analysis that included GENDER as a between-subjects factor; however, if this was non-significant then a further analysis was conducted without this factor. The total volume consumed/bodyweight^0.75^ and reward preference from reward consumption test were assessed by ANOVAs with between subjects factor of GENOTYPE (WT, PSW*^ICdel^*) and GENDER, and within subject factor of SESSION (Days 1 through 7), and the number of sessions taken to shape and train the mice in the SSRTT by unpaired two-tailed t-tests. SSRTT parameters were analysed by separate ANOVAs with between-subject factors of GENOTYPE and GENDER and within-subjects factor of STOP-SIGNAL POSITION (0 (mean BL), 10%, 20%, 30%, 40%, 50%, 60%, 70%, 80%, 90%) and STOP-PERFORMANCE (mean BL, 50% correct stopping). The effects of WAY163909 were analysed by ANOVA with between subject factors of GENOTYPE and GENDER and within-subjects contrasts factor of DOSE (vehicle, 0.1, 1 and 3 mg/kg), with vehicle as the planned contrasting level. For the analysis of c-Fos reactivity, separate two-way ANOVA were conducted for each brain region investigated, with between subject factors of GENOTYPE and DOSE (vehicle, 1 mg/kg WAY163909) were used. If a significant difference was found, then *post hoc* pairwise comparisons were performed using *t*-tests or Bonferroni tests, and adjusted for multiple comparisons. Criterion level of significance was set at the 0.05 level. All data are shown as mean ± standard error of the mean (S.E.M.).

## Funding

The Biotechnology and Biological Research Council UK (BB/J016756/1). The authors are members of Medical Research Council Centre for Neuropsychiatric Genetics and Genomics (G0801418).

## Supplementary Material

Davies_et_al-Supplementary_Information_ddz100
