# Supplementary material for: Prader–Willi syndrome imprinting centre deletion mice have impaired baseline and 5-HT2CR-mediated response inhibition
Source: Hum Mol Genet. 2019 May 14;28(18):3013–23. doi: 10.1093/hmg/ddz100 (PMC6737253; doi:10.1093/hmg/ddz100)
Supplement: Davies_et_al-Supplementary_Information_ddz100 [file davies_et_al-supplementary_information_ddz100.docx]

HMG-2019-D-00157

**PWS imprinting centre deletion mice have impaired baseline and 5HT_2C_R-mediated response inhibition**

Jennifer R Davies^1^, Lawrence S Wilkinson^1,2^, Anthony R Isles^1^ & Trevor Humby^2^*

1.Behavioural Genetics Group, MRC Centre for Neuropsychiatric Genetics and Genomics, Neuroscience and Mental Health Research Institute, Schools of Medicine^1^ & Psychology^2^, Cardiff University, Cardiff, CF14 4XN, UK.

*Corresponding authors:

Trevor Humby *Email* [HumbyT@cardiff.ac.uk](mailto:HumbyT@cardiff.ac.uk) *Tel.* ++44 (0)2920 876758

**Supplementary Information**

- Supplementary Materials and Methods Figure 1. Mouse stop-signal reaction time task (SSRTT) shaping and training.
- Supplementary Materials and Methods Figure 2. Calculation of the stop-signal reaction time (SSRT).
- Supplementary Results Table 1: Training and shaping measures
- Supplementary Results Table 2: Comparison of gender following WAY163909 treatment
- Supplementary Results Figure 1: Gender differences in WT and PWS*^ICdel^* mice in the Stop-signal reaction time task.

| **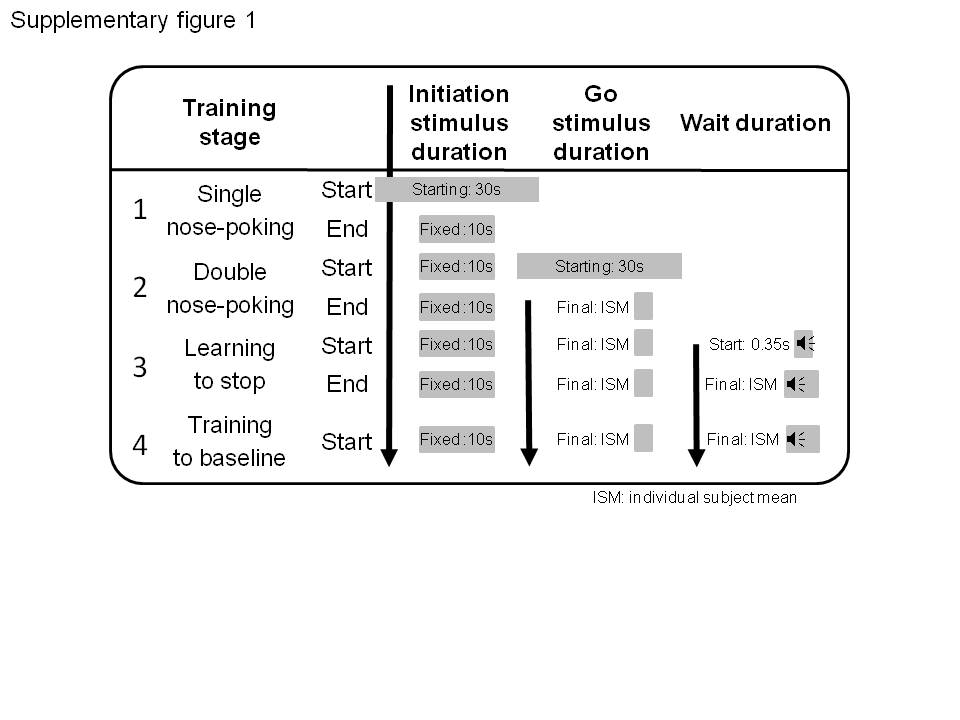** |
| --- |
| **Supplementary Materials and Methods Figure 1. Mouse stop-signal reaction time task (SSRTT) shaping and training.** Training to baseline involved shaping the mice to make nose-pokes at two locations in the stimulus array, an initiation response to the left and then a go response to the right-hand location, before then learning to withhold responding when an auditory stop-signal was presented. Each new aspect of the procedure was added during separate stages of training. Sessions were terminated after 100 trials had been presented or 20min had expired. Following initial shaping, in the first stage of training the mice were trained to make a single nose-poke to earn a reward, the ‘initiation response’, by gradually reducing the initiation stimulus duration from 30s to 10s. Once criteria performance was achieved in stage 1 (>70% trials completed) the mice were trained to make a rapid ‘go’ response between the two stimulus locations to earn reward (stage 2 of training). The go stimulus duration was initially set to 30s, and the duration was reduced to a value corresponding to the correct response latency for each individual subject, the go limited hold. Once at criteria performance (>80% correct going), a brief auditory stimulus was introduced in 20% of initiated trials (designated as ‘stop trials’) and the mice trained to withhold responding to the go response (stage 3 of training). The 0.3s long stop-signal was always presented at the start of the go response during training and baseline, i.e. 0% position relative to the individualised correct go reaction times of each subject. The duration that the mice were expected to wait for the reward delivery, the stop limited hold, was gradually increased to a value approximately equivalent to the go limited hold. Once the mice were at criteria stopping (>80% correctly stopped trials), the go and stop limited hold values were fixed (i.e. baseline performance) and the performance of each subject allowed to stabilise (stage 4). Once the animals had demonstrated stable performance at criteria (>70% trials initiated, and >80% correct go and stop trials), task manipulations such as moving the presentation time of the stop-signal, and assessing the effects of brain lesions and drugs were carried out. |

| 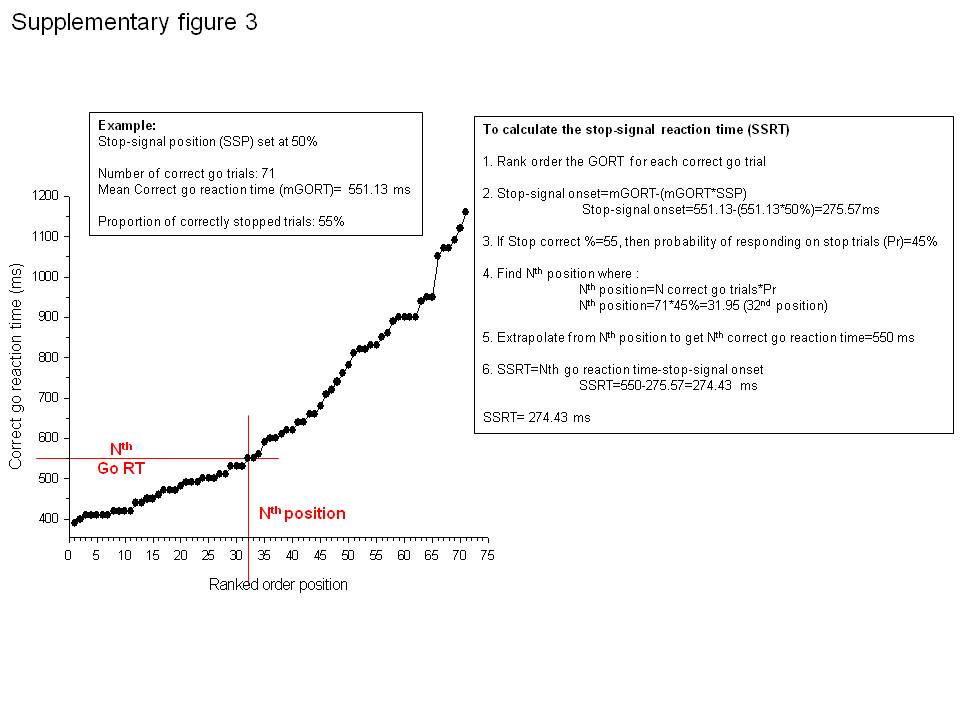 |
| --- |
| **Supplementary Materials and Methods Figure 2. Calculation of the stop-signal reaction time (SSRT).** An estimate for the SSRT, the time at which the stopping process terminated, was determined when subjects showed ~50% correct stopping (which equated to a 50% stop-signal position in C57Bl/6 mice), in order to ensure balanced contributions from underlying psychological and brain processes of going and stopping according to the predominant ‘race’ model of behavioral inhibition as assayed in the SSRTT (see Logan, 1994, Eagle *et al*., 2003). Using the methods of Eagle et al (2003), and Logan (1994), the SSRT was derived from the distribution of correct go reaction times (GoRT), based upon the probability of responding in stop trials (Pr). Thus, to calculate the SSRT, the N^th^ go reaction time was determined from the rank ordered correct go reaction times with onset time of the stop-signal (SSO) deducted. Thus, the SSRT=N^th^GoRT-SSO, where the N^th^GoRT is the value of the GoRT (in ms) for the N^th^ rank position of all correct go trials (N). The N^th^ rank position of go trials is the overall number of go trials (N) * the probability of responding in a stop trial (Pr): i.e. N*Pr. In the worked example above, data comes from a session in which the stop-signal was placed 50% into the correct go response, the mouse correctly completed 71 go trials, with a mean GoRT (mGoRT) of 551.13 ms and correctly withheld responding in stop trials 55% of the time. Therefore, to calculate the SSRT, the reaction times from the 71 correct go trials were ranked in order from lowest to highest (see graph). The N^th^ position was determined (N*Pr) from the probability of responding in a stop trial (Pr, 100%-55% correctly stopped trials=45%): 71 correct go trials*45%=32. The 32^nd^ GoRT=550 ms, by extrapolation from the 32^nd^ rank order position. The mean GoRT (mGoRT) in this session was 551.13 ms; therefore a 50% SSO was calculated as mGoRT-(mGoRT*50%), 551.13-(551.13*50%)=275.57 ms. Finally, SSRT was determined as the N^th^GoRT-SSO, 550-275.57=274.43 ms. SSRTs were calculated individually for each subject in each test session. |

**Supplementary Results Table 1: Training and shaping measures**

**
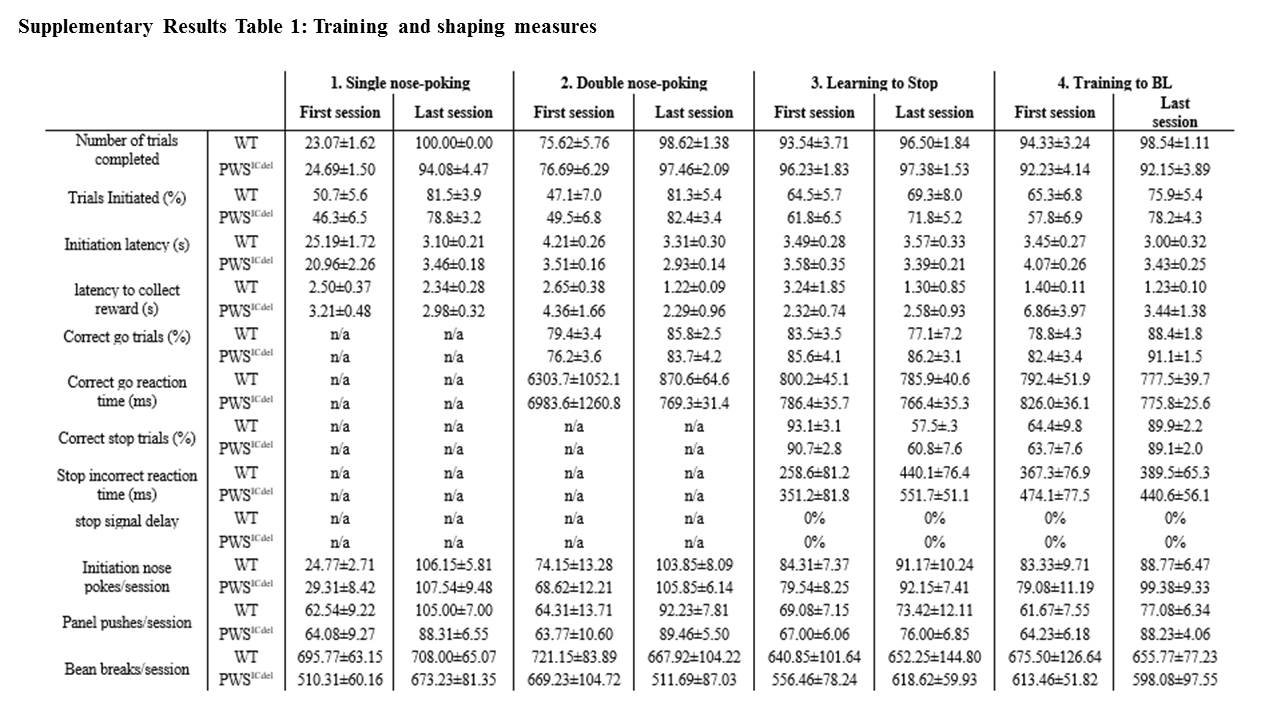
**

**Supplementary Results Table 2: Comparison of gender following WAY163909 treatment**

| 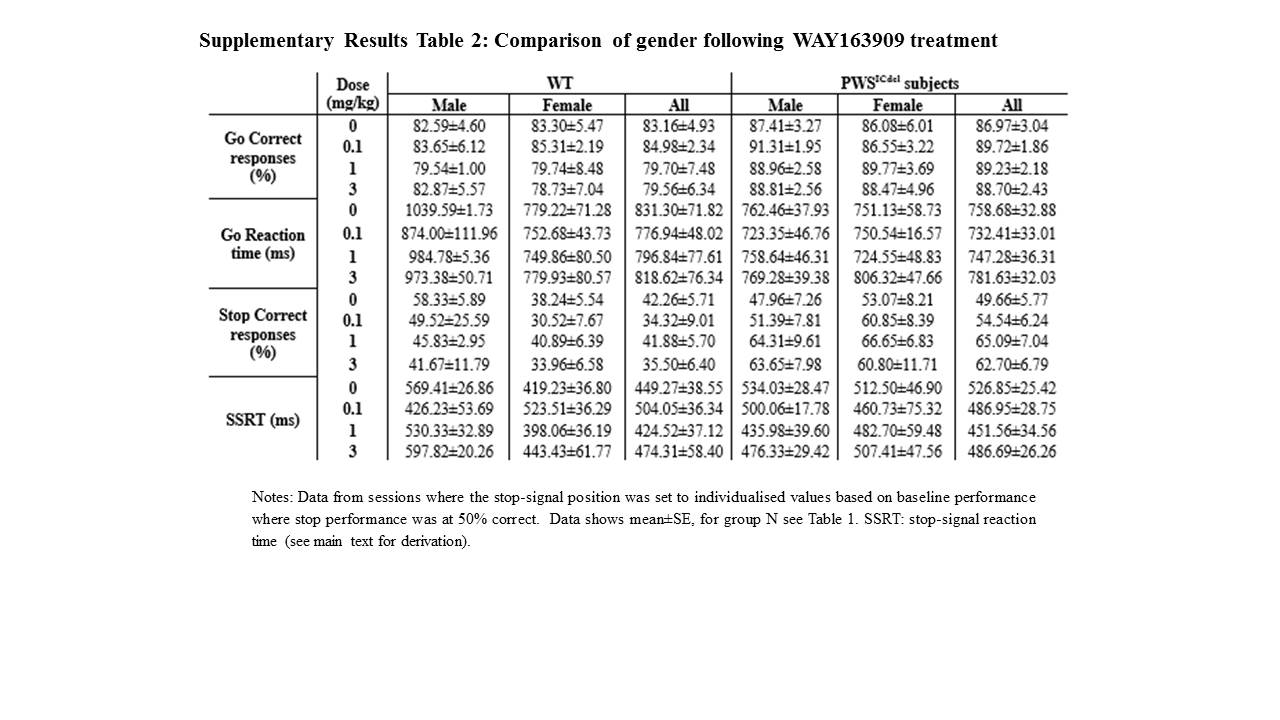 |
| --- |
| Notes: Data from sessions where the stop-signal position was set to individualised values based on baseline performance where stop performance was at 50% correct. Data shows mean±SE, for group N see Table 1. SSRT: stop-signal reaction time (see main text for derivation). |

| 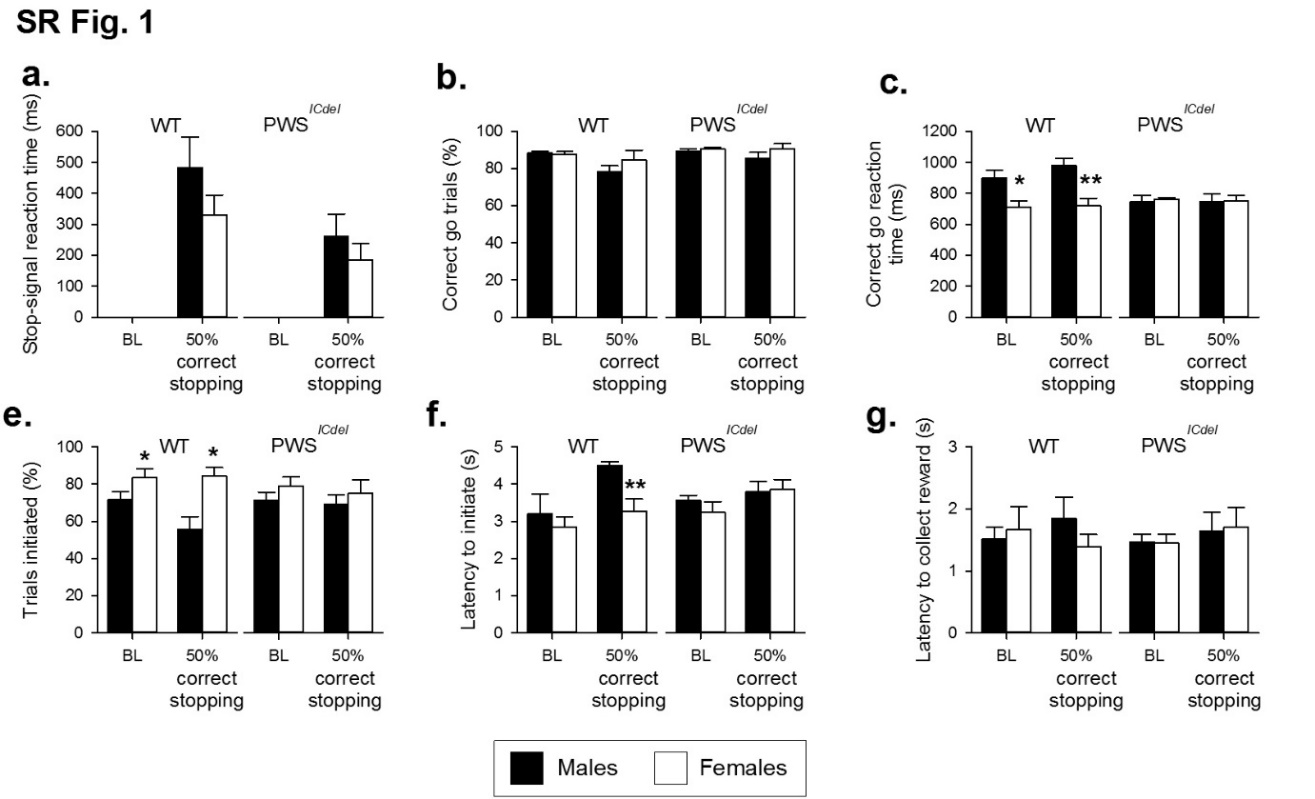 |
| --- |
| **Supplementary Results Figure 1: Gender differences in WT and PWS*^ICdel^* mice in the Stop-signal reaction time task.** Using the analysis of SSRTT performance at the point where competition between the go and stop responding was at its greatest where correct stopping was at 50±10% (see Main Text Figure 2), gender differences were examined. When stopping with 50% accuracy, there were no differences between WT or PWS*^ICdel^* male and female mice in terms of the stop-signal reaction time (a), proportion of correct trials (b) or for the latency to collect reward (g). WT female mice were quicker to make the go response (c) and completed more trials (e), at both baseline and when stopping with 50% accuracy, but there were no differences between male and female PWS*^ICdel^* mice for these measures. Female WT mice were also quicker than WT males to initiate a trial (f). Baseline data (BL: i.e, mean of the sessions immediately preceding each session where the stop-signal position was altered) when the stop-signal presentation were concurrent with the start of the go response (0%). For genotype/gender N see Table 1. Data are mean±SEM, * and ** denotes p<0.05 and **p<0.01 for significant difference between male and female mice, respectively. |
